# Smart Lies and Sharp Eyes: Pragmatic Artificial Intelligence for Cancer Pathology: Promise, Pitfalls, and Access Pathways

**DOI:** 10.3390/cancers18030421

**Published:** 2026-01-28

**Authors:** Mohamed-Amine Bani

**Affiliations:** Department of Medical Biology and Pathology, Gustave Roussy, 94800 Villejuif, France; mohamed-amine.bani@gustaveroussy.fr

**Keywords:** computational pathology, digital pathology, artificial intelligence, deep learning, biomarker prediction, validation, external validation, automation bias, stain normalization, low- and middle-income countries

## Abstract

**Background**: Whole-slide imaging and algorithmic advances have moved computational pathology from research to routine consideration. Despite notable successes, real-world deployment remains limited by generalization, validation gaps, and human-factor risks, which can be amplified in resource-constrained settings. **Content/Scope**: This narrative review and implementation perspective summarizes clinically proximate AI capabilities in cancer pathology, including lesion detection, metastasis triage, mitosis counting, immunomarker quantification, and prediction of selected molecular alterations from routine histology. We also summarize recurring failure modes, dataset leakage, stain/batch/site shifts, misleading explanation overlays, calibration errors, and automation bias, and distinguish applications supported by external retrospective validation, prospective reader-assistance or real-world studies, and regulatory-cleared use. We translate these evidence patterns into a practical checklist covering dataset design, external and temporal validation, robustness testing, calibration and uncertainty handling, explainability sanity checks, and workflow-safety design. **Equity Focus**: We propose a stepwise adoption pathway for low- and middle-income countries: prioritize narrow, high-impact use cases; match compute and storage requirements to local infrastructure; standardize pre-analytics; pool validation cohorts; and embed quality management, privacy protections, and audit trails. **Conclusions**: AI can already serve as a reliable second reader for selected tasks, reducing variance and freeing expert time. Safe, equitable deployment requires disciplined validation, calibrated uncertainty, and guardrails against human-factor failure. With pragmatic scoping and shared infrastructure, pathology programs can realize benefits while preserving trust and accountability.

## Simple Summary

Artificial intelligence (AI) is rapidly entering cancer pathology, promising faster, more reproducible diagnoses and new biomarkers that are invisible to the naked eye. Yet the same systems can mislead through shortcut learning, batch and stain drift, data leakage, or over-confident user interfaces that nudge clinicians into “automation bias.” Drawing on our International Conference on Contemporary Oncology 2025 presentation and a targeted review, we summarize what AI can reliably do today (detection, quantification, triage, and selected biomarker predictions), where it fails, and how to buy and validate tools safely. We also focus on practical pathways for low- and middle-income countries: choosing high-value use cases, minimizing compute and storage needs, building governance and training, and sharing validation assets across sites. Our goal is pragmatic: help pathology teams deploy AI that reduces variance and frees expert time, while guarding against the “smart lies” that can erode trust, equity, and patient outcomes.

## 1. Introduction

Cancer care is experiencing a pivotal transition in which digitization of pathology and the maturation of artificial intelligence (AI) could help address rising global need while also introducing new risks that demand careful governance. In 2022, there were an estimated ~20.0 million new cancer cases and 9.7 million cancer deaths worldwide, with the global annual incidence projected to reach ~35 million by 2050 [1,2]. Diagnostic capacity is a major bottleneck: nearly half of the world’s population lacks access to basic diagnostics, and workforce shortages in pathology persist even in well-resourced settings [3]. Within this context, whole-slide imaging (WSI) and computational pathology have moved from promise to practice. Digital pathology alone, however, does not change diagnostic reasoning; it primarily changes where and how slides are viewed and shared. Computational pathology adds a second layer, algorithmic measurement and decision support, that can standardize selected readouts, prioritize workload, and potentially reveal slide-level patterns linked to clinically relevant biology. Clinical adoption has accelerated, guided by consensus validation standards and multiple regulatory clearances for primary diagnosis [4,5,6,7,8,9,10].

The last decade has produced compelling demonstrations of what AI can add to surgical pathology, but the strength of evidence varies across use cases. In benchmark challenges and reader studies, algorithms have matched or exceeded pathologists on narrowly defined tasks (e.g., detection of lymph-node metastases in breast cancer) and, crucially, have improved human-reader performance when deployed as decision support [11,12,13]. Beyond detection, deep learning applied to routine hematoxylin–eosin (H&E) slides can recover prognostically and therapeutically relevant biology, including microsatellite instability (MSI) and other molecular signatures in colorectal cancer, hinting at a future in which morphology-derived biomarkers complement or triage more costly ancillary testing [14,15,16,17]. In immuno-oncology, automated, standardized scoring of immunohistochemistry (IHC), such as programmed death-ligand 1 (PD-L1) tumor proportion score (TPS), has shown analytical validity and potential clinical utility, addressing a recognized source of interobserver variability [18]. Importantly, computational pathology has also matured in non-oncologic settings, including quantitative assessment of chronic liver disease (e.g., non-alcoholic fatty liver disease and non-alcoholic steatohepatitis activity and fibrosis), algorithm-assisted interpretation of kidney biopsies and transplant pathology, and histology-based activity scoring in inflammatory bowel disease, providing a broader precedent for clinically meaningful, workflow-adjacent AI when ground truth and protocols are well defined [19,20,21].

Meanwhile, systematic scans of the commercial landscape suggest that only a minority of marketed digital pathology AI products are supported by peer-reviewed external validation, underscoring the need for transparent evidence frameworks and post-market performance monitoring [22].

At the same time, this progress has revealed how AI can mislead if development and evaluation are not aligned with clinical reality. Replicability and transportability remain fragile: results reported on internal test sets often degrade substantially when models are exposed to new laboratories, scanners, staining protocols, or patient populations [23]. Shortcut learning, batch effects, and hidden stratification can drive deceptively high in-distribution accuracy while encoding spurious correlations that fail under domain shift [9,24,25,26]. In this manuscript, we use the term “smart lies” to denote clinically plausible but incorrect model outputs produced by preventable technical and human-factor mechanisms, most commonly spurious correlations (shortcut learning), dataset leakage, domain shift, and miscalibrated confidence, whose presentation can further induce automation bias. Even well-performing tools can nudge clinicians toward error through automation bias unless user interfaces and workflows are deliberately engineered to support appropriate skepticism and fail-safes [27,28]. These “smart lies” are preventable but require disciplined dataset curation, clearly separated development/validation cohorts (at patient- and site-level), prospective reader studies in representative workflows, and continuous monitoring after deployment.

Equity considerations are central to this Special Issue on access to modern oncology in developing countries. Digital pathology can extend subspecialty expertise through teleconsultation, shorten turnaround times for critical diagnoses, and create data assets for local model tuning. However, without intentional design, AI risks widening disparities: infrastructure requirements, proprietary data silos, and licensing costs can hinder uptake in low- and middle-income countries (LMICs). International guidance now articulates practical guardrails for responsible AI in health, including validation on representative populations, data protection, transparency, and adaptive regulation, which are directly applicable to computational pathology [29,30]. Early LMIC implementations of digital pathology show feasibility but also highlight persistent barriers around connectivity, maintenance, workforce training, and sustainable financing; these must be addressed for AI benefits to materialize where the need is greatest [9,31,32,33,34,35]. Accordingly, the central question is not whether AI can work under ideal conditions, but how laboratories with heterogeneous pre-analytics and constrained resources can adopt high-value tools safely, verify performance locally, and sustain quality over time.

This article is a narrative review with an implementation perspective. To improve reproducibility, we performed a targeted literature search in PubMed/MEDLINE and Google Scholar (last searched: 9 January 2026), supplemented by forward/backward citation tracking of high-impact reviews, consensus guidelines, and pivotal validation studies. We prioritized publications from 2015 to 2026, with an emphasis on multi-institutional external validation, prospective/real-world workflow evaluations, and regulatory-cleared intended uses when available; preprints were considered only when directly relevant and clearly labeled as non-peer-reviewed. Inclusion focused on cancer pathology applications of whole-slide images, deployment science, and digital pathology implementations. Given heterogeneity in datasets, endpoints, and workflows, we did not perform a formal meta-analysis. We (i) summarize the current evidence for AI-enabled tasks in pathology that are closest to practice, explicitly distinguishing regulatory-cleared use, prospective deployments/reader-assistance studies, and retrospective or investigational reports; (ii) catalog common failure modes and present a pragmatic checklist to avoid “smart lies”; and (iii) propose an implementation pathway tailored to resource-limited settings that couples rigorous validation with equity-by-design. Our goal is to help laboratories, clinicians, and policymakers leverage digital pathology and AI to improve cancer outcomes, safely, reproducibly, and fairly.

## 2. The Digital/AI Inflection in Pathology

### 2.1. From Digitization to Decision Support

The inflection began with routine WSI adoption and quality-assured validation for primary diagnosis. As scanners, storage, and viewers matured into dependable clinical infrastructure, digital pathology primarily improved access and logistics, remote review, teleconsultation, sharing for multidisciplinary meetings, standardized viewing conditions, and archiving, without fundamentally changing the interpretive act itself. In parallel, algorithmic work moved from handcrafted features to convolutional networks and, more recently, to self-supervised and multimodal pre-training on very large slide corpora. This second layer, computational pathology, adds measurement and decision support: it can detect candidate regions, quantify predefined readouts, and prioritize cases, with the intended benefit of reducing variance and focusing expert attention [36,37]. Clinically, this progression shifted the question from whether AI can detect cancer on H&E to where AI most reliably adds value to patient care, namely, as targeted assistance embedded in the diagnostic workflow rather than as a stand-alone oracle [4,5,6,7,10,11,15]. Accordingly, “digital” and “AI” should be evaluated differently: digital pathology is an enabling platform with operational benefits, whereas AI is a clinical intervention whose value must be demonstrated by incremental improvements in accuracy, efficiency, standardization, or downstream testing.

### 2.2. Standards, Regulation, and Evidence

College of American Pathologists (CAP) WSI validation guideline updates, multiple U.S. Food and Drug Administration (FDA) 510(k) clearances for primary diagnosis, and In Vitro Diagnostic Medical Devices Regulation (IVDR) conformity for selected CE-marked tools now delineate a credible route from development to routine use. However, regulatory clearance and scientific evidence are not interchangeable: clearance typically establishes safety and intended performance within a defined use case, whereas local clinical value depends on fit with pre-analytics, case mix, workflows, and downstream decision pathways. At the same time, market scans show that comparatively few commercial products report peer-reviewed external validation or post-market performance, underscoring the need for transparent, updatable dataset/model documentation and mandatory local verification studies before go-live. To improve interpretability, evidence for AI tools should be communicated explicitly along an “evidence maturity” spectrum (e.g., retrospective internal testing, external multi-site validation, prospective reader-assistance or real-world impact studies, regulatory-cleared use) because the level of evidence determines how confidently a tool can be relied upon in routine care. In short, the regulatory door is open, but evidentiary discipline remains the rate-limiting step [4,5,6,7,14].

### 2.3. Data Scale and Learning Paradigms

Weak supervision on slide-level labels and multiple-instance learning enabled training at a clinical scale (tens of thousands of WSIs), while self-supervised pretraining further reduced annotation needs and improved sensitivity for subtle or rare findings. These advances also unlocked morphology-derived biomarker prediction directly from H&E. Nevertheless, domain shift, across institutions, stains, scanners, and time, remains a first-order failure mode. For this reason, performance claims should not be based on internal cross-validation alone; they should be supported by external testing on unseen sites and, when feasible, temporal validation that captures batch drift. External and temporal validation, with patient- and site-level splits and stratified reporting, is therefore mandatory for any claim of transportability [10,11,15,16,17]. Practically, laboratories should maintain a small, locally curated “challenge set” spanning common pre-analytic variation (stain intensity, section thickness, scanner family, and artifacts) to verify model behavior before deployment and after major changes.

### 2.4. Human Factors

Reader studies consistently show that AI assistance can raise accuracy, reduce interobserver variability, and shorten time-to-sign-out, but also that assistance can alter error profiles and induce automation bias if interfaces overstate certainty or reveal suggestions too early. Workflow configuration is therefore part of the intervention. Designs that have been tested in reader-assistance settings commonly include (1) an independent first read before AI overlays are revealed; (2) optional, reversible overlays that can be toggled on/off; (3) calibrated probability displays and uncertainty cues rather than binary prompts; and (4) explicit “override” capture when the pathologist disagrees, enabling audit and continuous improvement. Safe designs expose calibrated probabilities and reversible overlays, tether explanations to auditable evidence (e.g., masks and per-cell tables), and preserve an independent first read before AI hints are shown. These human-factor guardrails are as critical to clinical reliability as the underlying algorithmic performance [8,9,10,18]. In this sense, reliability is a property of the human-AI system, not the model alone, and implementation decisions (timing, user interface, and escalation rules) can determine whether AI reduces or amplifies error.

## 3. What AI Can Reliably Do Today (Clinically Adjacent Wins)

### 3.1. Detection and Triage

Across several narrowly defined, high-signal tasks, machine-learning systems now deliver consistent, reproducible gains when used as assistive tools rather than stand-alone diagnostics. Most supporting evidence comes from retrospective multi-site validation and reader studies, with fewer prospective workflow evaluations; regulatory-cleared intended uses exist for selected products and jurisdictions. Examples include detection of small tumor deposits in lymph nodes (e.g., breast sentinel nodes), where clinically implemented AI reduced reflex IHC usage and total costs without missed metastases, and reader assistance for difficult prostate biopsies, where AI support decreased requests for ancillary IHC and second opinions while maintaining accuracy. Similarly, robust performance has been shown for mitosis detection in multiple tumor types under domain shift (scanners/stains), for quality-control modules that flag out-of-focus areas, tissue folds, or incomplete coverage before sign-out, and for rare-event screening such as *Helicobacter pylori* on routine H&E in gastric biopsies. In prospective or real-world deployments, the clearest benefits appear as improved sensitivity at fixed specificity (or vice versa), reductions in downstream ancillary testing, and workload prioritization that pulls likely-positive or time-critical cases to the top of the queue, especially under sustained volume pressure [38,39,40,41,42,43,44].

### 3.2. Quantification

Where ground truth is protocolized and metrics are explicitly defined, AI-assisted quantification improves reproducibility and auditability while shortening reporting time. This is particularly evident for immunomarker scoring (e.g., PD-L1 TPS in lung cancer; Ki-67 in breast and neuroendocrine tumors; and hormone receptors/HER2 components), enumeration tasks (tumor-infiltrating lymphocytes, TILs), and morphometric features with guideline anchors (tumor budding and necrosis fraction). Multiple validation studies report tighter inter-observer agreement with algorithmic pre-segmentation and exportable masks/overlays that can be inspected, corrected when needed, and archived for audit trails; importantly, these tools align best with standardized pre-analytics and clearly written scoring standard operating procedures [44,45,46,47,48,49,50,51]. As for detection, most data are retrospective with selected prospective reader-assistance studies; therefore, clinical positioning is strongest as audited quantifiers that support, but do not replace, pathologist verification [52].

### 3.3. Morphology-Derived Surrogates for Molecular Features

For a subset of biomarkers with strong morpho-molecular coupling, deep learning on H&E can act as a fast prescreen to triage confirmatory testing. The most mature examples include microsatellite instability (MSI) prediction in colorectal cancer, where multi-site studies consistently report areas under the curve (AUCs) compatible with triage use (e.g., “rule-in” for reflex testing or prioritization when tissue is scarce), and *IDH* mutation status in gliomas, where whole-slide and multimodal models achieve high discrimination and can complement neuropathologist assessment [14,16,17,53]. *BRAF* V600E prediction shows promising results in several primaries (thyroid, melanoma, and colorectal), with external validations emerging [54,55], and promising tools are being developed for other actionable molecular alterations [56]. In all cases, these models should be positioned as clinically adjacent, accelerating or economizing the pathway, rather than as replacements for standard-of-care molecular/IHC assays. Accordingly, the most defensible intended use is triage or prioritization, with confirmatory testing remaining mandatory and with calibrated thresholds and abstention policies for low-confidence or out-of-distribution inputs.

### 3.4. Workflow Utilities

A second class of “reliable today” tools improves the process of pathology rather than the diagnostic endpoint. Stain and style normalization reduces inter-site color variation, aiding human and algorithmic readers; virtual re-staining (e.g., H&E ↔ trichrome or collagen surrogates) can support review and education and is being explored for fibrosis scoring; and pre-analytic quality-control modules raise early warnings about blur, stapling, tissue folds, or empty tiles before a case reaches sign-out. In operational pilots, case-level routing and triage have shown potential to shorten time-to-review by prioritizing likely positive or complex cases, though rigorous, prospective impact evaluations remain relatively scarce compared with detection or quantification tasks. Such utilities are safest when confined to “assistive boundaries,” with visible overlays, reversible effects, and explicit safeguards against hallucinated features in any image-to-image model. For image-to-image utilities in particular, the original slide must remain the diagnostic reference, and transformed views should be clearly labeled as supportive to avoid implicit over-trust [41,57,58,59].

### 3.5. Prognosis and Treatment-Response Prediction

Beyond detection and quantification, recent studies show that H&E-based deep learning can extract prognostic signals that complement stage and grade. Weakly supervised whole-slide models trained on unannotated WSIs predict disease-specific survival and identify histologic patterns associated with outcome, with performance maintained after external validation; more recent self-supervised transformers reported robust, attention-based survival stratification in multi-institutional cohorts [60,61,62]. When WSI features are fused with pathway-level transcriptomic representations, multimodal models further improve risk prediction, suggesting that morphology encodes clinically relevant host–tumor interactions that standard scores may miss. These results support the use of WSI-derived risk scores as adjuncts to conventional pathology rather than stand-alone classifiers, with local verification and calibration required prior to clinical adoption. Compared with detection/quantification, however, evidence is more heterogeneous and predominantly retrospective; therefore, clinical claims should be restricted to risk stratification support pending prospective utility evaluation and careful control of confounding.

A parallel line of work tackles treatment response. In non–small-cell lung cancer treated with immune checkpoint inhibitors (ICIs), H&E-based deep learning pipelines have been externally validated across nearly one thousand patients, showing association with response, progression-free survival, and overall survival at a level comparable to PD-L1; crucially, combining the AI score with PD-L1 improved stratification. Similar approaches in small-cell lung cancer report WSI-based prediction of both prognosis and therapeutic response, while in breast cancer, stromal morphology captured on routine slides predicts pathological complete response after neoadjuvant chemotherapy across multiple centers [63,64,65]. These approaches remain investigational in most settings and should be framed as hypothesis-generating or decision-support research until prospective impact is demonstrated.

An additional, clinically practical niche is WSI-based surrogates for multigene recurrence assays. Early peer-reviewed and preprint reports show that deep learning on H&E can estimate Oncotype-DX–like recurrence scores in hormone-receptor–positive early breast cancer, with promising agreement with the molecular assay across independent institutions [61,66]. While insufficient today to replace gold-standard testing, such tools could serve as prescreens when tissue is limited, to prioritize reflex testing, or to flag discordant morphology for multidisciplinary review.

### 3.6. Specimen Adequacy and Intraoperative Support

WSI-based algorithms also provide operational gains that matter for downstream precision oncology. Automated tumor-cell proportion (cellularity) estimation on H&E is now supported by multi-site validations and commercial-grade tools, helping determine whether blocks are suitable for biomarker testing and reducing variability in manual estimates. Early intraoperative studies, meanwhile, demonstrate that AI-assisted frameworks can aid frozen-section workflows, including margin assessment, and even generate virtual formalin-fixed paraffin-embedded (FFPE)-like images to stabilize interpretation; these applications remain investigational but illustrate credible near-term assistive roles [67,68,69]. Given the time-critical nature of intraoperative decisions, adoption requires especially conservative thresholds, explicit failure-mode handling, and prospective evaluation in representative workflows.

The clinical wins above share a common profile: a narrowly scoped task with clear and measurable ground truth; standardized pre-analytics and scoring protocols; prospective human-in-the-loop validation that demonstrates added value over routine practice; auditability via exportable overlays and case-level summaries; calibrated outputs that expose uncertainty; and an explicit intended-use statement aligned with regulatory expectations for software-as-a-medical-device (SaMD). Following contemporary reporting/validation guidance and local professional bodies’ AI position statements helps ensure that “assistive” remains truly assistive in routine care [70,71,72]. To make the distinction between routine-ready tools and investigational approaches explicit, we summarize major computational pathology applications by evidence level and current deployability in Table 1.

## 4. Beyond Narrow Tasks: Agents and Multimodal Models

As narrow, single-endpoint algorithms mature, attention is shifting to systems that coordinate multiple tools and reason over heterogeneous data. At present, most of these approaches remain investigational in pathology: the opportunity is real, but the evidence base is dominated by retrospective studies, technical demonstrations, or early pilots rather than routine, prospective clinical deployment as summarized in Table 1. Two trajectories are particularly salient for routine pathology practice: (i) agentic systems in which large language models (LLMs) orchestrate pathology workflows by calling validated tools and (ii) multimodal models that fuse WSI representations with textual and structured clinical information. Both directions promise broader utility than task-specific classifiers but also raise new requirements for governance, provenance, and prospective evaluation.

Agents for workflow orchestration, stain suggestions, and report drafting. In an agentic pattern, an LLM is not a diagnostic oracle but a controller that interprets the case context, queries the software for metadata, invokes validated image or quantification models, and synthesizes outputs into drafts for human verification. In practice, this can mean proposing evidence-based IHC panels when morphology and clinical metadata suggest specific differentials; pre-filling synoptic elements from audited quantifiers (e.g., tumor size, TILs, and necrosis fraction); or generating a first draft of the microscopic description while linking every statement to the underlying mask, measurement, or guideline citation. In this framing, agents function as workflow amplifiers that improve completeness and consistency, while leaving diagnostic judgment and accountability with the pathologist [73,74].

Multimodal models that fuse WSI and text for complex reasoning. Beyond orchestration, models that learn joint representations from histology and clinical text (clinical notes, gross description, IHC reads, radiology summaries) enable tasks that depend on cross-modal context. Typical architectures pair a self-supervised WSI encoder with a text encoder trained on pathology reports; cross-attention or late-fusion layers then learn associations between visual tokens and linguistic cues, such as the growth pattern, anatomic site, or biomarker status. Early results indicate consistent gains over unimodal baselines for survival modeling and treatment-response prediction and suggest that multimodal attention maps can surface human-readable rationales that complement heatmaps [75,76].

The most immediate constraints are therefore data governance and evaluation. Multimodal systems require linkable, de-identified corpora that cross departmental boundaries; consent language and data-sharing agreements must explicitly cover cross-modal use and provenance preservation. Benchmarking should move beyond discrimination to include calibration, subgroup equity, and end-to-end task success measured prospectively in routine workflows. For agentic systems, risk concentrates on error propagation: a single incorrect intermediate step (hallucinated differential, wrong retrieval, and miscalibrated model output) can be amplified downstream into a fluent but incorrect report draft. Mitigations should be operationally concrete: (i) enforce a “human-first” workflow where the pathologist’s independent impression is captured before AI suggestions are shown; (ii) require explicit human confirmation for any draft content imported into the final report; (iii) preserve immutable audit trails linking each generated sentence to its source (retrieved text, measurement, or overlay); and (iv) define abstention/escalation rules when inputs are incomplete, out-of-distribution, or when modality-specific uncertainty is high. Methodologically, promising directions include causal or counterfactual probes that test whether cross-modal associations are clinically meaningful rather than spurious; uncertainty estimation that exposes modality-specific confidence and triggers abstention or human escalation; and tool-using agents that default to audited quantifiers for any numeric claim and to guideline retrieval for any recommendation. Finally, feasibility will depend on deployability: edge-deployable encoders, efficient retrieval pipelines, and incremental fine-tuning schemes will determine whether these capabilities extend beyond a handful of well-resourced centers.

## 5. Where and Why AI Falls Short (“Smart Lies”)

Despite substantial progress in computational pathology, several recurring failure modes limit the reliability and transportability of current systems. These limitations are not merely technical curiosities; they can invert effect sizes, mask clinically important subgroups, and, if left unmanaged, undermine trust at deployment. Here we synthesize the most consequential issues and their practical implications for clinical translation.

A first source of error is dataset bias and hidden stratification, whereby aggregate metrics conceal poor performance in clinically meaningful subsets. When labels are coarse (e.g., slide-level cancer present/absent) but relevant subclasses differ in prevalence or visual phenotype, models may optimize for the majority class while systematically failing on rare or aggressive variants. This problem is exacerbated by weak supervision and proxy labels; high AUCs at the cohort level can coexist with substantial subgroup errors. In routine practice, this can manifest as confident misclassification of mucin-poor foci, intratumoral variants, or treatment-altered morphology, despite excellent headline metrics during development. Because such failures often emerge only after stratified audits (by grade, stage, histotype, treatment status, specimen type, or scanner), reporting should routinely include subgroup-level sensitivity/specificity and confidence intervals, not only global AUCs. Rigorous subgroup audits, error-slicing by stage/grade/site, and reporting of stratified metrics are therefore essential [26,77,78].

A second and pervasive challenge is data leakage and overly optimistic validation. In digital pathology, splitting data at the tile or patch level allows tiles from the same whole-slide image, or even the same patient, to appear in both development and test sets. This contaminates evaluation with near-duplicates, inflating accuracy and obscuring generalization gaps. Proper patient-level (or slide-level) splits, leakage checks, and external validation across institutions and scanners are necessary to obtain credible estimates of clinical performance. Practically, this requires enforcing patient-level separation, screening for duplicate slides/blocks and near-duplicate tiles, and documenting split logic in a reproducible manner (including temporal separation when drift is expected). Tooling that records predictions at tile, slide, and patient levels and that enforces cohort-aware splits can materially reduce this risk [25,79,80].

A third limitation concerns domain shift and brittleness to pre-analytic variation. Differences in fixation, processing, stain protocols, and scanner optics alter color and texture statistics in ways that confound models trained on a single site. While stain normalization and self-supervised pre-training on diverse corpora improve robustness, they do not eliminate shift-induced failures, particularly when pathology-specific cues are subtle [81,82,83,84]. Quantitatively, multi-site evaluations repeatedly report non-trivial external degradation, often on the order of several AUC points (approximately ~0.05–0.15) and/or sensitivity changes of ~5–20 percentage points depending on task, site, and thresholding strategy; in worst-case shifts, failure can be abrupt (“cliff edge”) rather than gradual [26]. Prospective evaluations across sites and temporal batches, coupled with continual performance monitoring, remain mandatory. Notably, similar shift-driven brittleness has been reported in computational pathology tasks beyond oncology, reinforcing that these risks are modality- and workflow-driven rather than disease-specific.

Fourth, interpretability surrogates are frequently misleading. Popular saliency and attribution maps can remain stable even when the underlying model is randomized, and apparent “explanations” may highlight background or slide artifacts rather than the morphologic features driving correct decisions. In the diagnostic setting, such brittle explanations risk over-trust. More reliable approaches, case-based reasoning with prototypical patches, feature ablation studies tied to pathologist-defined regions of interest, and prospectively specified model-critique checks are better aligned with pathology workflows [85,86,87]. For clinical routing or triage use cases, calibration is not optional: threshold selection, escalation policies, and “no-call/abstain” behavior should be pre-specified and re-verified after changes in stain, scanner, or software versions [88,89].

Fifth, modern deep networks are often miscalibrated: their probability estimates do not match the true likelihood of correctness, and overconfidence worsens under distribution shift. In human–AI teaming, poorly calibrated scores encourage automation bias (over-reliance on wrong suggestions) or, conversely, unjustified dismissal of helpful cues. Calibrating outputs (e.g., temperature scaling and ensembles), exposing uncertainty alongside predictions, and defining explicit abstention policies for low-confidence or out-of-distribution inputs attenuate these effects and make model outputs auditable [89,90,91].

Finally, human factors and governance determine real-world impact. Reader-assistance studies show that assistance can improve efficiency or sensitivity, but also that erroneous prompts may be followed uncritically, particularly under workload pressure. Safe deployment, therefore, requires prospectively specified intended-use statements, guardrails in the user interface (e.g., requiring a human estimate before revealing AI output), pre-registered impact metrics (diagnostic accuracy, time-to-sign-out, and equity across subgroups), and a maintenance plan for model updates and drift [91]. Recent regulatory guidance on predetermined change control plans for AI-enabled devices underscores that post-market learning must be anticipated rather than improvised. Within pathology departments, this translates into continuous quality management for models, analogous to IHC assay validation, with versioning, audit trails, and scheduled re-verification after scanner, stain, or software updates [72,92].

**Figure 1:**
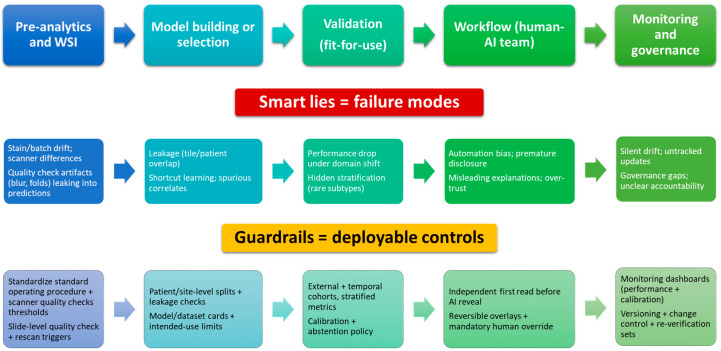
From “smart lies” to safe, clinically adjacent AI in pathology. A pragmatic blueprint linking common failure modes to deployable guardrails across the end-to-end lifecycle.

Taken together, these “smart lies” are actionable. Robust design (patient-level splits or leakage checks), diverse training and prospective, multi-site evaluation, calibrated and auditable outputs, and explicit human-factor safeguards can transform brittle prototypes into dependable clinical tools. The aim is not to replace pathologists, but to embed computational systems whose limitations are understood, measured, and mitigated so that incremental gains are reliable and reproducible in everyday practice. Key failure modes and corresponding deployment guardrails are summarized in Figure 1.

## 6. Implementer’s Validation and Deployment Blueprint

Translating computational pathology from promising prototypes to dependable clinical tools begins with an explicit intended-use statement and a pre-specified benefit–risk hypothesis. To avoid over-generalizing “reliable” performance, implementers should explicitly state the evidence tier supporting a use case (retrospective validation, prospective workflow deployment, or regulatory-cleared SaMD) and align the validation burden to the clinical risk and claim strength. Before model training, implementers should define the clinical decision to be supported, the target population and specimens, the workflow touchpoints (pre-screen, concurrent assist, or retrospective QA), and the primary and secondary endpoints that will indicate success. These endpoints should combine analytical performance (discrimination, calibration, and failure rate) with clinically meaningful effects, such as changes in reader accuracy, time-to-sign-out, reduction in ancillary testing, or equity of performance across demographic and site subgroups, and should be registered prospectively when formal evaluations are planned.

Data assembly must reflect the future use case rather than the convenience of available archives. Curating development cohorts from multiple laboratories, scanners, staining protocols, and time periods reduces shortcut learning and improves transportability. Ground truth should be obtained with rigorous, pathology-appropriate reference standards (e.g., multi-reader consensus with adjudication; immunohistochemistry or molecular confirmation for ambiguous foci), and labeling protocols should be documented and reproducible. Splitting must occur at the patient level (and, ideally, the site level for an external hold-out), with leakage checks to prevent near-duplicate tiles from entering both development and test sets. When longitudinal drift is plausible, a temporally separated test set is preferable to random sampling [25,26].

Internal testing is necessary but insufficient: prospective and truly external validation is the critical step in estimating real-world performance. External cohorts should be drawn from sites not represented in development, with pre-specified analysis plans and stratified reporting that surfaces hidden stratification (e.g., by tumor grade, size, treatment effect, or scanner family). Alongside discrimination metrics (AUC, sensitivity/specificity, and precision–recall), implementers should quantify calibration, for example, with reliability curves, Brier score, and expected calibration error, because probability estimates drive thresholds, triage policies, and human trust [26,80]. Domain shift should be reported quantitatively where possible, because external performance losses can be clinically meaningful (often on the order of several AUC points and/or several to tens of percentage points in sensitivity at fixed specificity, depending on task and shift), and these effects should be summarized by site, scanner family, and time period. Where outputs are used as triage gates, abstention policies for low-confidence or out-of-distribution inputs should be formalized and tested.

Reader-assistance studies connect analytical performance to clinical utility. Designs that require an independent first read before revealing AI suggestions reduce automation bias and better approximate intended practice. Outcomes should include accuracy and time effects, but also error-type shifts (e.g., misses converted to false positives), interobserver agreement, and equity across subgroups and sites. When the intended use is to replace or standardize a manual quantification (e.g., Ki-67 or PD-L1 scoring), analytical equivalence/equivocality bounds should be specified a priori and paired with qualitative usability and auditability assessments.

Safe deployment rests on robust systems engineering. Pre-analytics must be standardized, covering fixation, processing, staining, scanning, and quality control thresholds, and deviations should be visible to users and to the model monitoring stack. User interfaces should expose calibrated probabilities and uncertainty, offer reversible overlays, and capture discordance signals (instances where readers disagree with AI), which are invaluable for post-market surveillance and future model updates. Integration with the Laboratory Information System/Image Management System should support versioning, audit trails, and change control; any material change in hardware, software, or protocols should trigger re-verification against a locally curated challenge set, analogous to proficiency testing for IHC [4,6].

Governance aligns the above with regulatory expectations for software as a medical device. Documentation should include dataset and model cards, a statement of intended use and contraindications, a validation dossier with external and temporal testing, and a maintenance plan that specifies the key performance indicators, drift detection methods, update cadence, and rollback procedures. Where permissible, a predetermined change control plan can be used to delineate which post-market model updates are allowed without a new submission, provided that verification and validation steps and guardrails are defined in advance. Good machine learning practice principles further emphasize the performance of the human–AI team, transparency, and data/label management across the product lifecycle [29].

Equity and feasibility are integral, not afterthoughts, particularly in low- and middle-income settings. Validation cohorts should represent the populations and laboratories that will use the tool; if this is not possible at launch, implementers should plan for adaptive, locally tuned verification and threshold setting. Compute, storage, and bandwidth requirements should match local infrastructure, and licensing should provide data portability and exit clauses to avoid vendor lock-in. Training plans should target the whole team, including pathologists, lab technicians, and IT, because sustained performance depends on both human factors and technical maintenance. Finally, procurement should be tied to the delivery of evidence: vendors should provide access to validation reports, allow sandbox evaluation on local challenge sets, and commit to transparent monitoring dashboards after go-live.

Commercial SaMD solutions are not the only route. Many pathology departments are also exploring homebrew AI, locally developed or locally adapted models operated within a single institution under in-house test frameworks, extending the long-standing paradigm of homebrew IHC and special stains. Some clinically useful tools are too niche for industrial development. Homebrew AI is especially relevant for rare cancers or niche tasks that lack commercial incentives and for institutions seeking to tailor tools to local pre-analytics and populations. In resource-constrained settings, homebrew approaches can pair edge inference with intermittent synchronization, prioritize open-source stacks, and share anonymized challenge sets through professional societies to achieve external verification without costly vendor contracts. When pursued, homebrew AI must be run under laboratory-quality systems with manufacturer-like responsibilities for performance, documentation, and lifecycle management. Recent analyses argue that pathology is well-placed to adopt this model, provided that validation and oversight match clinical risk [93].

As for the regulatory landscape, in the European Union, Article 5(5) of the IVDR permits health institutions to manufacture and use in-house devices when specific conditions are met (e.g., use within the legal entity, quality management and technical documentation commensurate with risk, justification that patient needs cannot be met by an equivalent CE-marked device, and transparency on performance). Medical Device Coordination Group guidance clarifies that such in-house devices are exempt from third-party conformity assessment if used internally. However, the applicability of horizontal AI regulation (e.g., the EU AI Act) may depend on whether an AI system is placed on the market and on its functional classification; institutions should therefore document their legal rationale and monitor evolving guidance for “in-house” medical AI. This creates a pragmatic path for homebrew AI in European pathology, provided that institutions document intended use, analytical/clinical performance, and maintenance within their quality metrics [10,92,94].

In the United States, homebrew or laboratory-developed tests (LDTs) have historically operated under Clinical Laboratory Improvement Amendments with FDA enforcement discretion. Recent FDA moves to bring LDTs under tighter oversight and subsequent legal challenges culminating in a 2025 district-court decision striking down the LDT rule have created an evolving environment. Regardless of ongoing litigation, the practical takeaway for pathology is unchanged: laboratories that field homebrew AI must run them under rigorous quality metrics, with clear documentation of intended use, validation, and monitoring, and be prepared to adapt as federal policy settles [72,93,94].

Implementation responsibilities. Adopting or adapting open-source models does not absolve the laboratory of manufacturer duties. Departments should (i) retrain or fine-tune on locally representative data; (ii) conduct leakage-free internal testing and external or temporal validation; (iii) calibrate probabilities and set abstention thresholds; (iv) generate dataset/model cards and standard operating procedures (intended use, contraindications, failure modes); (v) version models and keep audit logs; (vi) institute drift monitoring with scheduled re-verification, and (vii) participate in external quality assessment where available. For quantitative tasks, homebrew AI should export auditable masks and per-cell tables, and analytical acceptance criteria should be pre-specified, mirroring IHC assay validation. Finally, to strengthen interpretability and prevent “smart lies,” departments should pre-define a minimum “monitoring bundle” (performance KPIs, calibration drift, failure/abstention rates, and subgroup dashboards) and link monitoring triggers to operational actions (threshold adjustment, retraining, rollback, or workflow changes).

## 7. An LMIC-Tailored Pathway for Digital and AI-Enabled Pathology

Expanding access to high-quality cancer diagnosis in LMICs requires a deployment model that is realistic about constraints yet ambitious about impact. The enabling argument is straightforward: whole-slide imaging (WSI) and computational pathology can extend subspecialty expertise across distance, standardize key measurements, and create local data assets for iterative improvement. However, digital pathology itself has distinct trade-offs (capital costs, maintenance, storage, connectivity, and workflow change), and AI adds additional layers of risk (domain shift, calibration drift, and human-factor failure), so benefits must be demonstrated with pragmatic endpoints before scaling. The countervailing realities, limited scanner fleets, variable power and bandwidth, fragmented information systems, and workforce shortages demand a phased approach that privileges reliability, interoperability, and affordability. A growing body of work from LMIC hospital settings and global health agencies converges on the same design principles: build the digital core first, verify benefits with pragmatic endpoints, and scale only when operations are stable. A minimal operational checklist for LMIC implementation (requirements and monitoring signals) is provided in Table 2.

A practical first phase focuses on telepathology and quality-assured digitization. This phase institutes WSI for selected, high-value specimen classes and second-opinion teleconsultation, with immediate gains not only for cancer diagnostics but also for non-cancer domains that commonly rely on remote expertise (e.g., infectious and inflammatory pathology, renal pathology, hematopathology, and transplant pathology) [95]. Image management is configured “offline-first” (local caching with store-and-forward synchronization) to tolerate intermittent connectivity, and consent/data-protection procedures are harmonized with institutional policies. Early assistance is confined to tasks with strong transportability and clear ground truth, such as pre-analytic quality check, safety-net detection/triage for rare but consequential findings, and robust quantification where staining and scanning are already standardized. Operational endpoints at this stage are time-to-diagnosis for teleconsults, avoiding rescans, and reduction in avoidable ancillary IHC when a second reader is readily available.

The second phase expands coverage from selected specimens to common tumor pathways while introducing audited quantifiers and simple workflow orchestration. Interoperability becomes a non-negotiable requirement: scanners and archives are normalized where possible, overlays and measurements are persisted as structured artifacts, and orders/results are exchanged with the LIS. Sites curate a local “challenge set” that mirrors their pre-analytics and case mix to verify transportability prior to go-live and after any material change (stain protocol, scanner firmware, or software update). Prospective monitoring dashboards track turnaround times, slide failure rates, and analytic indicators such as calibration and abstention rates, stratified by site and subgroup to surface hidden stratification early. At this phase, “success” is defined by measurable workflow impact (time saved, error types shifted, and ancillary tests avoided) rather than by headline AUC alone.

In the third phase, programs pilot morphology-derived triage for molecular testing, cautiously explore multimodal models that couple WSI with structured report elements for prognosis or treatment-response enrichment, and consider “homebrew” solutions for niche tasks that lack viable commercial products. Because these capabilities are typically supported by heterogeneous evidence (often retrospective, sometimes multi-site, rarely prospective in routine LMIC workflows), they should be framed as pilots with pre-specified stop/go criteria and explicit contraindications. To keep recurrent costs predictable, inference is preferentially run on modest on-premise servers with periodic synchronization (“edge inference”), using the cloud only where bandwidth and data-sovereignty constraints permit. Procurement is tied to evidence: vendors (or in-house developers) provide external/temporal validation summaries, allow sandbox evaluation on the local challenge set, and commit to transparent post-market monitoring. Financing plans explicitly model the total cost of ownership, maintenance, calibration, storage growth, and power conditioning and include exit clauses and data-portability guarantees to avoid lock-in.

Workforce and change management are as important as technology. Pathologists, biomedical scientists, and IT staff are trained together on pre-analytics, scanning, viewer use, and AI review, with clear standard operating procedures, versioned documentation, and escalation paths for urgent cases. Reader assistance is configured to preserve an independent first read before AI suggestions are revealed, overlays are reversible and auditable, and discordance signals (instances where the human reader disagrees with AI) are captured to feed local quality improvement. Regional partnerships amplify scarce expertise and create a practical route to external verification without prohibitive costs. Sites define a small set of operational key performance indicators (scanner uptime, rescans, and time-to-review), analytical indicators (discrimination, calibration, abstention rates, and error-type shifts), and equity indicators (performance stratified by hospital, sex, age, and tumor subtype). Any material change in staining, scanning, software, or workflow triggers a pre-defined re-verification on the local challenge set before returning to routine service. This “measure-as-you-scale” posture ensures that incremental gains are retained, risks are surfaced early, and scarce resources are spent on interventions that demonstrably improve access and patient-relevant outcomes [9,32,33,57,93].

## 8. Conclusions

Computational pathology has crossed from proof of concept to dependable, clinically adjacent assistance for clearly defined tasks. Deployed as decision support rather than replacement diagnostics, contemporary systems improve sensitivity for rare or easily overlooked findings, stabilize quantitative scoring for protocolized biomarkers, and streamline case prioritization under sustained workload pressure. Equally clear, however, are the mechanisms by which impressive internal results can unravel in practice: shortcut learning, pre-analytic and site shifts, miscalibrated probabilities, misleading explanation surrogates, and human-factor pitfalls such as automation bias. These “smart lies” are preventable. Treating algorithms as high-impact laboratory tests converts brittle prototypes into trustworthy tools.

The opportunity now extends beyond narrow endpoints. Foundation and multimodal models can reduce annotation burdens and capture cross-modal context for prognosis and treatment-response prediction, while agentic (LLM-plus-tools) workflows can orchestrate stain suggestions and synoptic drafting with full provenance capture. None of these advances relaxes the evidentiary bar; they intensify it. Future evaluations should report not only discrimination but also calibration, equity across clinically meaningful subgroups, and pragmatic endpoints such as changes in ancillary testing, time-to-diagnosis, and interobserver agreement. Governance must anticipate post-market learning through predetermined change control, dataset/model cards, and routine drift monitoring.

Access pathways matter in LMIC settings, as a phased approach that prioritizes telepathology, pre-analytic quality control, and a small set of robust assistance tools can yield immediate benefits while building the digital core required for later capabilities. Where commercial products are unavailable or misaligned with local needs, carefully governed homebrew AI offers a legitimate route to equitable deployment. With rigorous validation, thoughtful systems engineering, and equity-by-design procurement, computational pathology can scale the “sharp eyes” of our field while disarming the “smart lies,” improving the timeliness, consistency, and reach of cancer diagnostics.

## Figures and Tables

**Table 1:** Practical evidence level of AI applications in computational pathology.

Application Class	Typical Maturity in the Literature	How to Position in Practice *
**Detection and triage**	Strongest evidence: many retrospective multi-site validations; some prospective/real-world deployments and reader-impact studies; and some regulatory-cleared products exist	Clinically adjacent/deployment-ready as second reader or triage with local verification and monitoring
**Quantification**	Strong evidence when ground truth is standardized; frequent multi-site validations; some prospective workflow evaluations; and some regulatory-cleared tools exist	Clinically adjacent/deployment-ready for protocolized metrics; best as audited overlays with human review
**Workflow utilities**	Quality check/routing: moderate-to-strong retrospective evidence; limited prospective impact studies. Virtual staining: mostly technical/early clinical feasibility with higher risk of artifacts/hallucination-like errors	Deployable for quality check/routing with guardrails; virtual staining remains investigational except for constrained/validated uses
**Morphology-derived molecular surrogates**	Mostly retrospective multi-site validation; prospective clinical utility studies limited; and regulatory-cleared tools rare	Triage/reflex-testing aid only; not replacement for molecular/IHC assays
**Prognosis prediction**	Predominantly retrospective; external validation increasing; and prospective decision-impact evidence limited	Investigational/research-to-translation; consider as adjunct in studies and carefully monitored pilots
**Treatment-response prediction**	Emerging retrospective multi-site validation; prospective utility evidence early	Investigational; best framed as enrichment/stratification research rather than routine decision-making
**Intraoperative support**	Early feasibility studies; limited external/prospective evidence	Investigational; constrained pilots only
**Agents (LLM + tools)**	Early prototypes; major safety risks (hallucination/provenance loss) without strong guardrails	Not “reliable today”; near-future direction under strict human-in-the-loop + provenance constraints
**Multimodal foundation models**	Early research; mostly retrospective; and evaluation standards still evolving	Near-future research direction; no routine deployment yet

* Regulatory-cleared tools and prospective/real-world evaluations vary by jurisdiction, product, and intended use.

**Table 2:** LMIC deployment checklist for digital and AI pathology.

Step	What You Must Have	What You Track
1. Scope	Narrow intended use (telepathology/quality check/detection/quantification); written contraindications	Adoption by use case; override/discordance rate
2. Pre-analytics	Basic standard operating procedure for fixation/staining/scanning + slide quality check rules	Quality check failures; rescan rate; and stain drift signals
3. Infrastructure	Offline-first image storage/viewing; power/maintenance plan	Scanner uptime; backlog; and turnaround time
4. Local verification	Small local “challenge set” reflecting local case mix	Pass/fail after updates; and drift flags
5. Workflow safety	Independent first read before AI; reversible overlays; human override	Reading time; error-type shifts; and automation-bias indicators
6. Integration	Case identification linkage + versioning; store overlays/metrics	Traceability (% cases with version recorded); missing artifacts
7. Training	Team training (pathologists + lab + IT); escalation rules	Completion; user-reported incidents
8. Monitoring and change control	Defined update policy + re-verification triggers	Calibration/abstention trends; rollback events

## Data Availability

No new data were created or analyzed in this study.

## Abbreviations

The following abbreviations are used in this manuscript:

AI	Artificial intelligence
AUC	Area under the receiver operating characteristic curve
CAP	College of American Pathologists
CE	Conformité Européenne (European Conformity) marking
FDA	Food and Drug Administration
FFPE	Formalin-fixed paraffin-embedded
H&E	Hematoxylin and eosin
IHC	Immunohistochemistry
IVDR	In Vitro Diagnostic Medical Devices Regulation
LDT	Laboratory-developed test
LLMs	Large Language Model
LMICs	Low- and middle-income country(ies)
MSI	Microsatellite instability
SaMD	Software as a medical device
TPS	Tumor proportion score
PD-L1	Programmed death-ligand 1
WSI	Whole-slide imaging

## References

[1] Bizuayehu H.M., Ahmed K.Y., Kibret G.D., Dadi A.F., Belachew S.A., Bagade T., Tegegne T.K., Venchiarutti R.L., Kibret K.T., Hailegebireal A.H. (2024). Global Disparities of Cancer and Its Projected Burden in 2050. JAMA Netw. Open.

[2] Bray F., Laversanne M., Sung H., Ferlay J., Siegel R.L., Soerjomataram I., Jemal A. (2024). Global Cancer Statistics 2022: GLOBOCAN Estimates of Incidence and Mortality Worldwide for 36 Cancers in 185 Countries. CA. Cancer J. Clin..

[3] Fleming K.A., Horton S., Wilson M.L., Atun R., DeStigter K., Flanigan J., Sayed S., Adam P., Aguilar B., Andronikou S. (2021). The Lancet Commission on Diagnostics: Transforming Access to Diagnostics. Lancet.

[4] CAP Launches Update to Whole Slide Imaging (WSI) Guideline. https://newsroom.cap.org/news-by-topic/cap-launches-update-to-whole-slide-imaging--wsi--guideline/s/7a6e394a-e659-42a0-8c2a-30811189cb45.

[5] Roche Receives FDA Clearance on Its Digital Pathology Solution for Diagnostic Use. https://diagnostics.roche.com/global/en/news-listing/2024/roche-receives-fda-clearance-on-its-digital-pathology-solution-for-diagnostic-use.html.

[6] Validating Whole Slide Imaging for Diagnostic Purposes in Pathology. https://www.cap.org/protocols-and-guidelines/cap-guidelines/current-cap-guidelines/validating-whole-slide-imaging-for-diagnostic-purposes-in-pathology.

[7] Masjoodi S., Anbardar M.H., Shokripour M., Omidifar N. (2025). Whole Slide Imaging (WSI) in Pathology: Emerging Trends and Future Applications in Clinical Diagnostics, Medical Education, and Pathology. Iran. J. Pathol..

[8] Jain E., Patel A., Parwani A.V., Shafi S., Brar Z., Sharma S., Mohanty S.K. (2024). Whole Slide Imaging Technology and Its Applications: Current and Emerging Perspectives. Int. J. Surg. Pathol..

[9] Bessen J.L., Alexander M., Foroughi O., Brathwaite R., Baser E., Lee L.C., Perez O., Gustavsen G. (2025). Perspectives on Reducing Barriers to the Adoption of Digital and Computational Pathology Technology by Clinical Labs. Diagnostics.

[10] van Diest P.J., Flach R.N., van Dooijeweert C., Makineli S., Breimer G.E., Stathonikos N., Pham P., Nguyen T.Q., Veta M. (2024). Pros and Cons of Artificial Intelligence Implementation in Diagnostic Pathology. Histopathology.

[11] Bulten W., Balkenhol M., Belinga J.-J.A., Brilhante A., Çakır A., Egevad L., Eklund M., Farré X., Geronatsiou K., Molinié V. (2021). Artificial Intelligence Assistance Significantly Improves Gleason Grading of Prostate Biopsies by Pathologists. Mod. Pathol..

[12] Ehteshami Bejnordi B., Veta M., Van Diest P.J., Van Ginneken B., Karssemeijer N., Litjens G., Van Der Laak J.A.W.M. (2017). the CAMELYON16 Consortium. Diagnostic Assessment of Deep Learning Algorithms for Detection of Lymph Node Metastases in Women With Breast Cancer. JAMA.

[13] Pati P., Jaume G., Ayadi Z., Thandiackal K., Bozorgtabar B., Gabrani M., Goksel O. (2023). Weakly Supervised Joint Whole-Slide Segmentation and Classification in Prostate Cancer. Med. Image Anal..

[14] Li H., Qin J., Li Z., Ouyang R., Chen Z., Huang S., Qin S., Huang Q. (2025). Systematic Review and Meta-Analysis of Deep Learning for MSI-H in Colorectal Cancer Whole Slide Images. NPJ Digit. Med..

[15] Sheng H., Zhang Y., Cong R., Wang S., Yang D., Cui Z., Huang X., Chen R., Liu J., Ke W. (2025). Deep-Learning Based Colorectal Cancer Pathological Analysis with Hyperspectral Light Field Microscopy. iScience.

[16] Yamashita R., Long J., Longacre T., Peng L., Berry G., Martin B., Higgins J., Rubin D.L., Shen J. (2021). Deep Learning Model for the Prediction of Microsatellite Instability in Colorectal Cancer: A Diagnostic Study. Lancet Oncol..

[17] Kather J.N., Pearson A.T., Halama N., Jäger D., Krause J., Loosen S.H., Marx A., Boor P., Tacke F., Neumann U.P. (2019). Deep Learning Can Predict Microsatellite Instability Directly from Histology in Gastrointestinal Cancer. Nat. Med..

[18] Plass M., Olteanu G., Dacic S., Kern I., Zacharias M., Popper H., Fukuoka J., Ishijima S., Kargl M., Murauer C. (2025). Comparative Performance of PD-L1 Scoring by Pathologists and AI Algorithms. Histopathology.

[19] Rubin D.T., Kubassova O., Weber C.R., Adsul S., Freire M., Biedermann L., Koelzer V.H., Bressler B., Xiong W., Niess J.H. (2025). Deployment of an Artificial Intelligence Histology Tool to Aid Qualitative Assessment of Histopathology Using the Nancy Histopathology Index in Ulcerative Colitis. Inflamm. Bowel Dis..

[20] Van Loon E., Callemeyn J., Roufosse C. (2023). Automating Kidney Transplant Rejection Diagnosis: A Simple Solution for a Complex Problem?. Clin. Kidney J..

[21] Lemley K.V. (2025). A Brief Review of Some Artificial Intelligence Methods in Nephrology. Pediatr. Nephrol..

[22] Matthews G.A., McGenity C., Bansal D., Treanor D. (2024). Public Evidence on AI Products for Digital Pathology. NPJ Digit. Med..

[23] Wagner S.J., Matek C., Shetab Boushehri S., Boxberg M., Lamm L., Sadafi A., Winter D.J.E., Marr C., Peng T. (2024). Built to Last? Reproducibility and Reusability of Deep Learning Algorithms in Computational Pathology. Mod. Pathol..

[24] Yamashita R., Long J., Banda S., Shen J., Rubin D.L. (2021). Learning Domain-Agnostic Visual Representation for Computational Pathology Using Medically-Irrelevant Style Transfer Augmentation. IEEE Trans. Med. Imaging.

[25] Bussola N., Marcolini A., Maggio V., Jurman G., Furlanello C. (2021). AI Slipping on Tiles: Data Leakage in Digital Pathology. Proceedings of the Pattern Recognition. ICPR International Workshops and Challenges.

[26] McGenity C., Clarke E.L., Jennings C., Matthews G., Cartlidge C., Freduah-Agyemang H., Stocken D.D., Treanor D. (2024). Artificial Intelligence in Digital Pathology: A Systematic Review and Meta-Analysis of Diagnostic Test Accuracy. NPJ Digit. Med..

[27] Straw I. (2020). The Automation of Bias in Medical Artificial Intelligence (AI): Decoding the Past to Create a Better Future. Artif. Intell. Med..

[28] Romeo G., Conti D. (2025). Exploring Automation Bias in Human–AI Collaboration: A Review and Implications for Explainable AI. AI Soc..

[29] World Health Organization (2021). Ethics and Governance of Artificial Intelligence for Health: WHO Guidance.

[30] Wong E.Y.T., Verlingue L., Aldea M., Franzoi M.A., Umeton R., Halabi S., Harbeck N., Indini A., Prelaj A., Romano E. (2025). ESMO Guidance on the Use of Large Language Models in Clinical Practice (ELCAP). Ann. Oncol..

[31] del Valle A.C. (2025). Leveraging Digital Pathology and AI to Transform Clinical Diagnosis in Developing Countries. Front. Med..

[32] Daniel M., Nowak K., Vajpeyi R., Clarke B., Evans A., Carment-Baker C., Weiser K., Martin M., Girard N., Fyfe K. (2025). From Microscopes to Monitors: Unique Opportunities and Challenges in Digital Pathology Implementation in Remote Canadian Regions. Diagnostics.

[33] Coudry R.A., Assis E.A.C.P., Frassetto F.P., Jansen A.M., da Silva L.M., Parra-Medina R., Saieg M. (2024). Crossing the Andes: Challenges and Opportunities for Digital Pathology in Latin America. J. Pathol. Inform..

[34] Aifuobhokhan J., Olatokun T.P., Ekechi C.C., Oladeji J., Olajide O.O., Ogunjinmi A., Popoola A., Fuwape O.D., Genevieve O.-A.O. (2025). Artificial Intelligence in Cancer Diagnosis: A Scoping Review of Global Innovation and African Implementation. World J. Adv. Res. Rev..

[35] Clarke B., Carment-Baker C., Bruce C., Hanna K., Yousef G.M. (2025). Large Scale Implementation of DP for Clinical Diagnoses: Experience, Challenges, and Lessons Learned. Crit. Rev. Clin. Lab. Sci..

[36] Nicke T., Schäfer J.R., Höfener H., Feuerhake F., Merhof D., Kießling F., Lotz J. (2025). Tissue Concepts: Supervised Foundation Models in Computational Pathology. Comput. Biol. Med..

[37] Hosseini M.S., Bejnordi B.E., Trinh V.Q.-H., Chan L., Hasan D., Li X., Yang S., Kim T., Zhang H., Wu T. (2024). Computational Pathology: A Survey Review and the Way Forward. J. Pathol. Inform..

[38] van Dooijeweert C., Flach R.N., ter Hoeve N.D., Vreuls C.P.H., Goldschmeding R., Freund J.E., Pham P., Nguyen T.Q., van der Wall E., Frederix G.W.J. (2024). Clinical Implementation of Artificial-Intelligence-Assisted Detection of Breast Cancer Metastases in Sentinel Lymph Nodes: The CONFIDENT-B Single-Center, Non-Randomized Clinical Trial. Nat. Cancer.

[39] Blilie A., Mulliqi N., Ji X., Szolnoky K., Boman S.E., Titus M., Gonzalez G.M., Asenjo J., Gambacorta M., Libretti P. (2025). Artificial Intelligence-Assisted Prostate Cancer Diagnosis for Reduced Use of Immunohistochemistry. Commun. Med..

[40] Gao G., Wei Z., Pei F., Du Y., Liu B. (2025). Deep Learning for Smartphone-Aided Detection System of *Helicobacter Pylori* in Gastric Biopsy. Sci. Rep..

[41] Jurgas A., Wodzinski M., D’Amato M., van der Laak J., Atzori M., Müller H. (2024). Improving Quality Control of Whole Slide Images by Explicit Artifact Augmentation. Sci. Rep..

[42] Khor L.Y., Neo C.C., Prathaban K., Choa E., Quah W.K., Lum E.N., Chen R., Heng S.Y., Koh V.C., Seow J.X. (2025). Deep Learning Model for Automated Detection of *Helicobacter Pylori* and Intestinal Metaplasia on Gastric Biopsy Digital Whole Slide Images. Am. J. Clin. Pathol..

[43] Campanella G., Chen S., Singh M., Verma R., Muehlstedt S., Zeng J., Stock A., Croken M., Veremis B., Elmas A. (2025). A Clinical Benchmark of Public Self-Supervised Pathology Foundation Models. Nat. Commun..

[44] Gessain G., Lacroix-Triki M. (2025). Computational Pathology for Breast Cancer: Where Do We Stand for Prognostic Applications?. Breast.

[45] Ito H., Yoshizawa A., Terada K., Nakakura A., Rokutan-Kurata M., Sugimoto T., Nishimura K., Nakajima N., Sumiyoshi S., Hamaji M. (2024). A Deep Learning–Based Assay for Programmed Death Ligand 1 Immunohistochemistry Scoring in Non–Small Cell Lung Carcinoma: Does It Help Pathologists Score?. Mod. Pathol..

[46] Knudsen B.S., Jadhav A., Perry L.J., Thagaard J., Deftereos G., Ying J., Brintz B.J., Zhang W. (2024). A Pipeline for Evaluation of Machine Learning/Artificial Intelligence Models to Quantify Programmed Death Ligand 1 Immunohistochemistry. Lab. Investig..

[47] Yosofvand M., Khan S.Y., Dhakal R., Nejat A., Moustaid-Moussa N., Rahman R.L., Moussa H. (2023). Automated Detection and Scoring of Tumor-Infiltrating Lymphocytes in Breast Cancer Histopathology Slides. Cancers.

[48] Van Rijthoven M., Aswolinskiy W., Tessier L., Balkenhol M., Bogaerts J.M.A., Drubay D., Blesa L.C., Peeters D., Stovgaard E.S., Lænkholm A.-V. (2025). Tumor-Infiltrating Lymphocytes in Breast Cancer through Artificial Intelligence: Biomarker Analysis from the Results of the TIGER Challenge. medRxiv.

[49] Öztürk S.K., Bokhorst J.-M., Baumann E., Sheahan K., van de Velde C.J.H., Marijnen C.A.M., Hospers G.A.P., Doukas M., Vieth M., Lugli A. (2025). Exploring Intratumoral Budding in Colorectal Cancer Using Computational Pathology: A Biopsy-Based Evaluation. Mod. Pathol..

[50] Bokhorst J.-M., Nagtegaal I.D., Zlobec I., Dawson H., Sheahan K., Simmer F., Kirsch R., Vieth M., Lugli A., Van Der Laak J. (2023). Semi-Supervised Learning to Automate Tumor Bud Detection in Cytokeratin-Stained Whole-Slide Images of Colorectal Cancer. Cancers.

[51] Campanella G., Kumar N., Nanda S., Singi S., Fluder E., Kwan R., Muehlstedt S., Pfarr N., Schüffler P.J., Häggström I. (2025). Real-World Deployment of a Fine-Tuned Pathology Foundation Model for Lung Cancer Biomarker Detection. Nat. Med..

[52] Volynskaya Z., Mete O., Pakbaz S., Al-Ghamdi D., Asa S.L. (2019). Ki67 Quantitative Interpretation: Insights Using Image Analysis. J. Pathol. Inform..

[53] Karabacak M., Ozkara B.B., Mordag S., Bisdas S. (2022). Deep Learning for Prediction of Isocitrate Dehydrogenase Mutation in Gliomas: A Critical Approach, Systematic Review and Meta-Analysis of the Diagnostic Test Performance Using a Bayesian Approach. Quant. Imaging Med. Surg..

[54] Ma Y., Jamdade S., Konduri L., Sailem H. (2025). AI in Histopathology Explorer for Comprehensive Analysis of the Evolving AI Landscape in Histopathology. NPJ Digit. Med..

[55] Wang C.-W., Muzakky H., Lee Y.-C., Lin Y.-J., Chao T.-K. (2023). Annotation-Free Deep Learning-Based Prediction of Thyroid Molecular Cancer Biomarker BRAF (V600E) from Cytological Slides. Int. J. Mol. Sci..

[56] Integrated Histopathologic Modeling of Detailed Tumor Subtypes and Actionable Biomarkers|bioRxiv. https://www.biorxiv.org/content/10.1101/2025.08.14.670351v1.

[57] Deman F., Broeckx G., Declercq S., Degotte Q., Raymaekers J., Salgado R., Dendooven A. (2025). Practical Implementation of AI in a Non-academic, Non-commercial Pathology Laboratory: Real World Experience and Lessons Learned. Histopathology.

[58] Voon W., Hum Y.C., Tee Y.K., Yap W.-S., Nisar H., Mokayed H., Gupta N., Lai K.W. (2023). Evaluating the Effectiveness of Stain Normalization Techniques in Automated Grading of Invasive Ductal Carcinoma Histopathological Images. Sci. Rep..

[59] Rakha E.A., Toss M., Shiino S., Gamble P., Jaroensri R., Mermel C.H., Chen P.-H.C. (2021). Current and Future Applications of Artificial Intelligence in Pathology: A Clinical Perspective. J. Clin. Pathol..

[60] Jiang X., Hoffmeister M., Brenner H., Muti H.S., Yuan T., Foersch S., West N.P., Brobeil A., Jonnagaddala J., Hawkins N. (2024). End-to-End Prognostication in Colorectal Cancer by Deep Learning: A Retrospective, Multicentre Study. Lancet Digit. Health.

[61] Garberis I., Gaury V., Saillard C., Drubay D., Elgui K., Schmauch B., Jaeger A., Herpin L., Linhart J., Sapateiro M. (2025). Deep Learning Assessment of Metastatic Relapse Risk from Digitized Breast Cancer Histological Slides. Nat. Commun..

[62] Yang Z., Wei T., Liang Y., Yuan X., Gao R., Xia Y., Zhou J., Zhang Y., Yu Z. (2025). A Foundation Model for Generalizable Cancer Diagnosis and Survival Prediction from Histopathological Images. Nat. Commun..

[63] Rakaee M., Tafavvoghi M., Ricciuti B., Alessi J.V., Cortellini A., Citarella F., Nibid L., Perrone G., Adib E., Fulgenzi C.A.M. (2025). Deep Learning Model for Predicting Immunotherapy Response in Advanced Non−Small Cell Lung Cancer. JAMA Oncol..

[64] Zhang Y., Yang Z., Chen R., Zhu Y., Liu L., Dong J., Zhang Z., Sun X., Ying J., Lin D. (2024). Histopathology Images-Based Deep Learning Prediction of Prognosis and Therapeutic Response in Small Cell Lung Cancer. NPJ Digit. Med..

[65] Bidard F.-C., Gessain G., Bachelot T., Frechin L., Vincent-Salomon A., Drubay D., Lemonnier J., Walter T., Penault-Llorca F., Martin A.-L. (2025). Identifying Patients With Low Relapse Rate Despite High-Risk Estrogen Receptor-Positive/Human Epidermal Growth Factor Receptor 2-Negative Early Breast Cancer: Development and Validation of a Clinicopathologic Assay. J. Clin. Oncol..

[66] Lee G., Lee J., Kwak T.-Y., Kim S.W., Kwon Y., Kim C., Chang H. (2025). Assessing the Risk of Recurrence in Early-Stage Breast Cancer through H&E Stained Whole Slide Images. Sci. Rep..

[67] L’Imperio V., Capitoli G., Cazzaniga G., Mannino M., Bono F., Seminati D., Eloy C., Pinto J., Rocco E.G., Fassan M. (2025). The Routine Use of a Digital Tool for the Tumor Cell Fraction Quantification in Molecular Pathology: An International Validation of QuANTUM. Pathol.-J. Ital. Soc. Anat. Pathol. Diagn. Cytopathol..

[68] Hatanaka K.C., Nishino K., Yokose T., Tanaka H., Motoi N., Taguchi K., Tamai Y., Hirai T., Yabuki Y., Hatanaka Y. (2025). Tumor Cell Proportion Assessment in Advanced Non-Squamous Non-Small Cell Lung Cancer Tissue Samples in Real-World Settings in Japan: The ASTRAL Study. Diagnostics.

[69] Özkan A., Schröder K.-M., Bronsert P., Franz J., Glienke M., Sigle A., Beck J., Werner M., Gratzke C., Straehle J. (2025). Validation of Artificial Intelligence–Enhanced Stimulated Raman Histopathology for Intraoperative Margin Assessment During Robot-Assisted Radical Prostatectomy: Preliminary Results from the ROBOSPEC Study. Eur. Urol. Open Sci..

[70] RCPath-AI-Position-Statement-2022.Pdf. https://www.rcpath.org/static/90e5e248-4ad3-4d61-8247223f9faffc80/RCPath-AI-position-statement-2022.pdf.

[71] Collins G.S., Moons K.G.M., Dhiman P., Riley R.D., Beam A.L., Van Calster B., Ghassemi M., Liu X., Reitsma J.B., van Smeden M. (2024). TRIPOD+AI Statement: Updated Guidance for Reporting Clinical Prediction Models That Use Regression or Machine Learning Methods. BMJ.

[72] FDA (2025). Guidances with Digital Health Content.

[73] Ahmed F., Yang L., Jaroensri T., Sellergren A., Matias Y., Hassidim A., Corrado G.S., Webster D.R., Shetty S., Prabhakara S. (2025). PolyPath: Adapting a Large Multimodal Model for Multislide Pathology Report Generation. Mod. Pathol..

[74] Alzaid E., Pergola G., Evans H., Snead D., Minhas F. (2024). Large Multimodal Model-Based Standardisation of Pathology Reports with Confidence and Its Prognostic Significance. J. Pathol. Clin. Res..

[75] Ding T., Wagner S.J., Song A.H., Chen R.J., Lu M.Y., Zhang A., Vaidya A.J., Jaume G., Shaban M., Kim A. (2025). A Multimodal Whole-Slide Foundation Model for Pathology. Nat. Med..

[76] Lu M.Y., Chen B., Williamson D.F.K., Chen R.J., Zhao M., Chow A.K., Ikemura K., Kim A., Pouli D., Patel A. (2024). A Multimodal Generative AI Copilot for Human Pathology. Nature.

[77] Oakden-Rayner L., Dunnmon J., Carneiro G., Re C. (2020). Hidden Stratification Causes Clinically Meaningful Failures in Machine Learning for Medical Imaging. Proceedings of the ACM Conference on Health, Inference, and Learning.

[78] Shahamatdar S., Saeed-Vafa D., Linsley D., Khalil F., Lovinger K., Li L., McLeod H.T., Ramachandran S., Serre T. (2024). Deceptive Learning in Histopathology. Histopathology.

[79] Dolezal J.M., Kochanny S., Dyer E., Ramesh S., Srisuwananukorn A., Sacco M., Howard F.M., Li A., Mohan P., Pearson A.T. (2024). Slideflow: Deep Learning for Digital Histopathology with Real-Time Whole-Slide Visualization. BMC Bioinform..

[80] Arun S., Grosheva M., Kosenko M., Robertus J.L., Blyuss O., Gabe R., Munblit D., Offman J. (2025). Systematic Scoping Review of External Validation Studies of AI Pathology Models for Lung Cancer Diagnosis. NPJ Precis. Oncol..

[81] Xu C., Sun Y., Zhang Y., Liu T., Wang X., Hu D., Huang S., Li J., Zhang F., Li G. (2025). Stain Normalization of Histopathological Images Based on Deep Learning: A Review. Diagnostics.

[82] Homeyer A., Geißler C., Schwen L.O., Zakrzewski F., Evans T., Strohmenger K., Westphal M., Bülow R.D., Kargl M., Karjauv A. (2022). Recommendations on Compiling Test Datasets for Evaluating Artificial Intelligence Solutions in Pathology. Mod. Pathol..

[83] Lin S., Zhou H., Watson M., Govindan R., Cote R.J., Yang C. (2025). Impact of Stain Variation and Color Normalization for Prognostic Predictions in Pathology. Sci. Rep..

[84] Michielli N., Caputo A., Scotto M., Mogetta A., Pennisi O.A.M., Molinari F., Balmativola D., Bosco M., Gambella A., Metovic J. (2022). Stain Normalization in Digital Pathology: Clinical Multi-Center Evaluation of Image Quality. J. Pathol. Inform..

[85] Adebayo J., Gilmer J., Muelly M., Goodfellow I., Hardt M., Kim B. Sanity Checks for Saliency Maps. Proceedings of the 32nd Conference on Neural Information Processing Systems (NeurIPS 2018).

[86] MacDonald S., Foley H., Yap M., Johnston R.L., Steven K., Koufariotis L.T., Sharma S., Wood S., Addala V., Pearson J.V. (2023). Generalising Uncertainty Improves Accuracy and Safety of Deep Learning Analytics Applied to Oncology. Sci. Rep..

[87] Wehkamp K., Krawczak M., Schreiber S. (2023). The Quality and Utility of Artificial Intelligence in Patient Care. Dtsch. Ärztebl. Int..

[88] Romanchikova M., Thomas S.A., Dexter A., Shaw M., Partarrieau I., Smith N., Venton J., Adeogun M., Brettle D., Turpin R.J. (2022). The Need for Measurement Science in Digital Pathology. J. Pathol. Inform..

[89] Ji X., Salmon R., Mulliqi N., Khan U., Wang Y., Blilie A., Olsson H., Pedersen B.G., Sørensen K.D., Ulhøi B.P. (2025). Physical Color Calibration of Digital Pathology Scanners for Robust Artificial Intelligence–Assisted Cancer Diagnosis. Mod. Pathol..

[90] Kwon D. (2025). How Artificial Intelligence Is Transforming Pathology. Nature.

[91] Rosbach E., Ganz J., Ammeling J., Riener A., Aubreville M. (2025). Automation Bias in AI-Assisted Medical Decision-Making Under Time Pressure in Computational Pathology 2024. Bildverarbeitung für die Medizin.

[92] Aldea M., Salto-Tellez M., Marra A., Umeton R., Stenzinger A., Koopman M., Prelaj A., Kehl K.L., Gilbert S., Leßmann M.-E. (2025). ESMO Basic Requirements for AI-Based Biomarkers in Oncology (EBAI). Ann. Oncol..

[93] Calderaro J., Morement H., Penault-Llorca F., Gilbert S., Kather J.N. (2025). The Case for Homebrew AI in Diagnostic Pathology. J. Pathol..

[94] Marketing Submission Recommendations for a Predetermined Change Control Plan for Artificial Intelligence-Enabled Device Software Functions; Guidance for Industry and Food and Drug Administration Staff; Availability. https://www.federalregister.gov/documents/2024/12/04/2024-28361/marketing-submission-recommendations-for-a-predetermined-change-control-plan-for-artificial?utm_source=chatgpt.com.

[95] Shean R.C., Rets A.V. (2025). Digital Pathology in Hematopathology: From Vision to Deployment. Int. J. Lab. Hematol..

